# Application and Analysis of Improved Fuzzy Comprehensive Evaluation Method in Goodwill Evaluation and Intangible Asset Management

**DOI:** 10.1155/2022/2235542

**Published:** 2022-09-09

**Authors:** Mengyue Xu

**Affiliations:** University of Toronto, Ontario, Mississauga L5L1C6, Canada

## Abstract

In order to improve the effect of goodwill evaluation and intangible asset management, this paper combines the improved fuzzy comprehensive evaluation method to construct an intelligent algorithm. In order to consider the many influencing factors of intangible asset prices and their own influence, this experiment adopts the ARDL model for analysis. Based on the traditional LSTM model, this paper innovatively introduces sparse principal component analysis for feature dimensionality reduction and, on the other hand, adds a convolutional neural network to the model, which is suitable for feature extraction. In addition, this paper constructs a goodwill evaluation and intangible asset management model based on the improved fuzzy comprehensive evaluation method. The research shows that the improved fuzzy comprehensive evaluation method proposed in this paper has a very good application effect in goodwill evaluation and intangible asset management.

## 1. Introduction

The capital value of intangible assets can be determined through the evaluation of intangible assets. Intangible assets can be valued as human shares in the process of group formation, listing, merger, and joint venture of an enterprise. Moreover, an accurate assessment of the value of intangible assets can provide an important protection for the company's intellectual property rights and proprietary technologies. At the same time, it can protect the company from having a value compensation standard when it is infringed, and based on this, it can ask the infringer for infringement damages, so as to avoid huge losses to the company [1]. The evaluation of the value of intangible assets by business owners can not only determine the value of assets [2]. In addition, information about the enterprise can also be obtained and provided to managers. Although the management of intangible assets of enterprises has gradually taken shape in recent years, the managers of enterprises are not clear about how many intangible assets an enterprise has and the value of the intangible assets, which can easily form a blind spot in enterprise asset management. After asset evaluation, the true value of assets can be fully revealed, which is more conducive to managers to make appropriate business plans according to the situation of the enterprise, and can also enhance the confidence of investors [3]. Second, intangible assets with clear value can generate economic benefits in economic activities, enabling enterprises to use externally for paid or mortgage loans to obtain funds when there is a problem of capital turnover. Furthermore, the evaluation of intangible assets can also reflect the influence and development of the brand in the industry and region, and understand the value of the corporate brand [4]. The development and management of the brand and the utilization of resources within the enterprise can be further understood by evaluating the value of intangible assets. Therefore, by adjusting the business plan of the enterprise through the choice of consumers among the industries, it is possible to obtain the operation plan and target of the next cycle and strive to maximize the profit of the enterprise [5].

From the perspective of the social function of asset appraisal, the social function of asset appraisal is to provide value scales for asset business and capital markets. Most of the current asset appraisal projects involve corporate restructuring or property rights changes, such as corporate mergers and acquisitions, listings, and sino-foreign joint ventures. The evaluation conclusion is an important basis for the asset business parties to conclude the transaction. If the capital price is inaccurate, it will not only mislead the allocation of resources, and reduce the efficiency of social resource allocation, but also cause misunderstandings in property rights transactions and various capital businesses, damage the legitimate rights and interests of the parties involved, and affect the security, prosperity, and stability of the capital market [6].

The analytic hierarchy process (AHP) is a method that combines quantitative and qualitative analysis methods for problems that cannot be quantitatively researched by establishing mathematical models and data, and follows the sequence of decomposition first and synthesis later, step by step, to deal with some complex problems, so as to obtain satisfactory decision-making results [7]. Therefore, AHP is also widely used in the evaluation of intangible assets. In the evaluation of machinery and equipment, the analytic hierarchy process is introduced to improve the new rate. Based on the purpose of determining the weight, the analytic hierarchy process is used to construct a judgment matrix, the weight and order of the required indicators are obtained, and the corresponding weight of each indicator is clarified. Combined with specific standards, the assignment of each influencing factor is completed to ensure the accuracy of the index system and avoid the influence of subjective factors [8]. In the process of implementing the construction of the data asset evaluation system, it is first necessary to analyze the relevant factors that affect the value of the data assets and combine the AHP to complete the construction of the final evaluation system. At the same time, it is necessary to ensure that the evaluation system can cover the corresponding costs and applications of the data assets. [9]. We calculate the main weights of different evaluation indicators and the corresponding target layer, design specific operation methods, and verify the operability of the evaluation system with examples [10]. The influencing factors and related indicators of intangible assets are obtained through the expert group, and the relative importance of each factor is obtained by using the analytic hierarchy process [11]. In the research of intangible assets of network database, the value evaluation of database assets is completed by the income method and the analytic hierarchy process, a certain degree of correction is carried out, and then, the weight of each asset is used to complete the evaluation of intangible assets [12]. With the help of the AHP, the evaluation and analysis of the goodwill value of the household appliance industry are carried out, and the factor model that affects the goodwill value is constructed to achieve the effect of revising the evaluation results of the traditional income method and improve the accuracy of the evaluation results [13]. In asset evaluation of all walks of life, especially intangible asset evaluation, the combination of AHP and income method has been widely recognized.

As far as the value transfer of the intangible assets of enterprises is concerned, it is generally expressed as external investment and transfer and sale. This transfer must be premised on the profitability of intangible assets. At this point, the focus of both parties in the transaction is on the function of the intangible asset, and the price of the intangible asset is determined accordingly. The way intangible assets play a role is obviously different from that of tangible assets [14].

Intangible assets do not have material entities and cannot be directly sensed by people. They are invisible assets, but such assets are often attached to certain entities. Physical entities such as equipment and production lines play their role, trademark rights are reflected through product quality, goodwill is embedded in the overall asset portfolio of the enterprise, and the value of intangible assets is materialized in the depth and breadth of tangible assets, which determines the degree of intangible assets. Therefore, the transfer price of intangible assets must fully consider the scope of intangible assets used to “arm” tangible assets [15].

The principles of economics tell us that the organic combination of people, wealth, and things is the source of asset appreciation. In the primitive capital accumulation process of capitalism, if an enterprise achieves assets of several hundred million, it requires the efforts of several generations. In modern economic society, the value added of intangible assets is on the rise. The value rapidly increases [16]. Intangible assets are like catalysts in chemical reactions, and levers in mechanical physics, accelerating and amplifying the effects of tangible assets. The high efficiency of the use value of intangible assets can bring future excess returns to enterprises [17].

The present value of income of intangible assets is to measure the actual value of intangible assets from the perspective of future expected income, which is beneficial to enterprises in the process of transferring intangible assets. It is more objective to observe and confirm the value of intangible assets in a certain period of time in the future, but the present value method of income inevitably has strong subjective judgment factors [18]. At present, this method mainly measures the transfer price of intangible assets, that is, the foreign investment and transfer business of intangible assets. Generally, the transfer price is greater than the book cost. For the original enterprise, if it is a transfer sale, the transfer price is the transfer price, and the original book cost is the transfer cost; if it is an external investment, the transfer price is the investment cost, and the value-added part (transfer price—book cost) is converted into enterprise capital reserve [19].

This paper combines the improved fuzzy comprehensive evaluation method to conduct research on goodwill evaluation and intangible asset management, and to improve the evaluation effect of intangible assets.

## 2. Intangible Asset Evaluation Algorithm

The full name of the ARDL model is the autoregressive distributed lag model. It includes the dependent variable, the independent variable, and the lag term of the independent variable. The model does not require that the variables must be of the same order, both I (0) and I (1) can be used, and the model can directly test the short-term and long-term relationships between the dependent variable and the independent variable. As long as the ordinary least square (OLS) assumption is satisfied, the model can be directly estimated. In order to consider the many influencing factors of intangible asset prices and their own influence, this experiment adopts the ARDL model for analysis.

The structure of the *ARDL*(*p*, *q*_1_, *q*_2_, ⋯, *q*_*k*_) model is as follows:

(1) $$\begin{array}{c} f\left(L,P\right)y_{t}=\overset{k}{\underset{i=1}{\sum }}\beta_{i}\left(L,q_{i}\right)x_{it}+dw\;t+ut. \end{array}$$

Among them,

(2) $$\begin{array}{c} f\left(L,P\right)=1-\phi_{1}L-\phi_{2}L^{2}-\ldots -\phi_{p}L^{p}, \end{array}$$

(3) $$\begin{array}{c} \beta_{i}\left(L,q_{i}\right)=1-\beta_{i1}-\beta_{i2}L^{2}-\ldots -\beta_{iq_{i}}L^{q_{i}}. \end{array}$$

In formulas (1)–(3), *p* is used to represent the lag order of *y*_*t*_, and *q*_*i*_ is the lag order of *x*_*it*_, *i*=1,2,3, ⋯, *k*. The lag operator *L* is defined as follows: *Ly*_*t*_=*y*_*t*−1_, *w*_*t*_ represents a vector with *d* rows and 1 column.

Regarding the determination of the lag order of the variable, the modified AIC criterion can be used. The criterion has strong theoretical advantages, and at the same time, the dummy variable with the minimum value is input to solve the problem of outliers. In regression, using these dummy variables can improve the reliability of the model.

**Figure 1:**
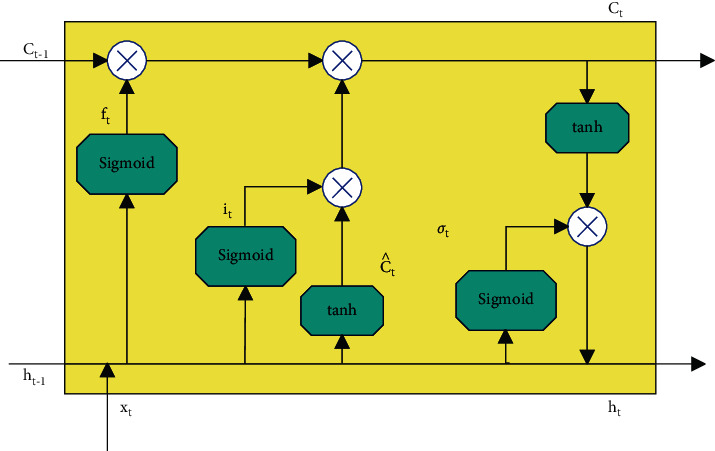
Schematic diagram of LSTM memory cell structure.

To solve the “long dependency problem” of recurrent neural networks, long short-term memory neural networks (LSTMs) were proposed. It is the most commonly used model in recursion and a variant of the recurrent neural network. Compared with the recurrent neural network, the LSTM method increases the memory cells used to form the processing. Based on the “long dependency problem,” coupled with the research and modification of many scholars, the more common LSTM structure now is the introduction of “gate.” The “gate” structure can select the most effective feature among many features for processing, so as to achieve the purpose of controlling the flow of information. A schematic diagram of the basic structure of LSTM is shown in Figure 1.

The structure of the recurrent neural network is a chain connection. The obtained information accepts the input of the previous node, outputs the current node state, and transmits it to the next node for input again. As can be seen from Figure 1, on the whole, the structure of LSTM is the same as that of the recurrent neural network, but the internal input and output have undergone major changes, and the number of transfer states has changed. Each storage unit of LSTM consists of a forget gate, an input gate, and an output gate. The functions of the three gates are to adjust the state of the transmitted information. In the process of transmission, the function of the forget gate can be summarized as “forget the unimportant and leave only the important.” The forget gate controls which information of the previous cell state *C*_*t*−1_ needs to be left and which needs to be forgotten through calculation. The sigmoid function exists as a gated state unit. When the function value is 0, all information is discarded, and when the function value is 1, all information is left. The wf and bf represent the weights and biases of the forget gate.

(4) $$\begin{array}{c} f_{t}=\sigma \left(wf\cdot \left[h_{t-1},x_{t}\right]+bf\right). \end{array}$$

Next, the input gate corresponds to the selection memory stage, and the function of this stage is to selectively “remember” the incoming information. Those who feel important information can occupy a larger proportion, and the unimportant information can be appropriately discarded. The gate control signal of the input gate is controlled by the tanh function. The calculation formula of this stage is as follows:

(5) $$\begin{array}{c} it=\sigma \left(wf∙\left[h_{t-1,}xt\right]+b_{i}\right)\\ \hat{C}t=\tan\;h\left(W_{c}\cdot \left[h_{t-1},xt\right]+b_{c}\right)\\ Ct=f_{t}*Ct-1+it*\hat{C}t. \end{array}$$

The last structure of the memory is the output gate structure, which corresponds to the output stage of the model. This stage mainly controls which information obtained will be regarded as the output of the current state. This process is controlled by the tanh function, which is equivalent to scaling the cell state obtained in the previous stage. The calculation formula of the entire output gate structure is as follows:

(6) $$\begin{array}{c} \sigma_{t}+\sigma \left(w_{o∙}\left[h_{t}-1,xt\right]+b0\right)\\ h_{t}=\sigma t*\;\tan\;h\left(Ct\right). \end{array}$$

The above is the basic model structure and calculation process of the LSTM model. The model needs to update the parameters in the empirical process, and the update process follows the gradient descent rule and the backpropagation rule.

Based on the traditional LSTM model, this paper innovatively introduces the sparse principal component analysis (SPCA) for feature dimension reduction on the one hand and adds a convolutional neural network to the model on the other hand. The convolutional neural network is suitable for feature extraction, and it is mainly used to deal with image classification problems in the early stage of its development. For example, it inputs a picture into the machine, and the machine tells us the category of this picture (such as flowers and people), and the convolutional neural network classifies by grabbing the distinctive category features in the picture. Nowadays, some studies also use it on one-dimensional time-series forecasting problems. In this paper, the convolutional layer is added to the traditional LSTM model, which can discover the data features more deeply. Using this network structure to train the prediction model can also obtain better prediction results.

### 2.1. Sparse Principal Component Analysis

Considering that some of the economic characteristic indicators that affect the price level of intangible assets selected in this paper are not completely independent of each other, such as oil and gold prices, there may be a certain correlation, resulting in redundant information between each characteristic. The correlation between variables may lead to problems such as the unstable structure of the model and heavy training burden. In this paper, SPCA is used for feature dimensionality reduction. Relatively speaking, although the principal component analysis can extract features, sometimes the extracted features are difficult to explain. By adding a penalty function, SPCA can sparse the principal component coefficients and highlight important components, which is conducive to interpretation.

The SPCA model is essentially an optimization problem, and the objectives to be optimized are as follows:

(7) $$\begin{array}{c} \min_{A,B}{\left\|{X-XBA}^{T}\right\|}_{F}^{2}\lambda \overset{k}{\underset{i=1}{\sum }}{\left\|\beta_{i}\right\|}^{2}\overset{k}{\underset{i=1}{\sum }}\lambda_{1,i}{\left\|\beta_{i}\right\|}_{1}\\ s.t.A^{T}A=Ik. \end{array}$$

In formula (10), *X* represents the collected data matrix, A and B are the coordinate blocks obtained by the coordinate descent method, and the initial value is calculated according to the load obtained by the principal component analysis. *β*_*i*_ is the load obtained from the solution, *p* is the number of variables, and *n* is the number of samples. Regarding the value of *λ*, if *n* ≤ *p* is in the dataset, *λ* > 0 is required; otherwise, *λ*=0 is taken. *λ*_1,*i*_ is a positive parameter, used to control the parameter *β*_*i*_.

Next, one of the two coordinate blocks is fixed, the other coordinate block is solved, and then, the solution is exchanged. The first *k* principal components obtained are denoted as *Y*_*i*_=*Xvi*, *i*=1,2, ⋯, *k*, and the obtained sparse principal components are used for fitting. The optimization problem (10) is transformed into *k* independent elastic net problems, and the formula is shown in

(8) $$\begin{array}{c} \min_{v\in R}\frac{1}{2}{\left\|X\beta -y_{i}\right\|}_{2}^{2}+\lambda {\left\|\beta \right\|}_{1},i=1,2,\cdots ,k. \end{array}$$

In the process of solving the above formula, since each problem is a standard lasso problem, it is possible to separately solve a subproblem first and then solve the remaining subproblems in the same way. After solving, the sparse load can be obtained, that is, *β*_*i*_ in formula (10). Multiplying the sparse loading matrix with the initially collected sample data matrix can get a new sample set after dimensionality reduction.

Regarding the coordinate descent method used to solve the lasso problem, the method is simple and easy to implement. The main principle is to solve only along a certain coordinate direction and fix other directions. The problem transformation of the solution process is as follows:

(9) $$\begin{array}{c} \beta_{i}^{*}={\text{argmin}}_{\beta }\left\{\overset{n}{\underset{j=1}{\sum }}X_{ji}^{2}\beta_{i}^{2}-\left(\overset{n}{\underset{j=1}{\sum }}X_{ji}^{2}r_{j}^{\left(i\right)}\right)\beta_{i}+\lambda \left|\beta_{i}\right|\right\}. \end{array}$$

Among them, *r*_*j*_^(*i*)^=*y*_*j*_ − Σ_*k*≠*i*_*x*_*jk*_*β*_*k*_.

Before solving problem (9), proposition 1 needs to be given first.

**Proposition 1 .**

∀*a* ∈ *R*, and*λ* > 0, *Sλ*(*a*)/*b* represents the minimum point of the function 1/2*bz*^2^ − *az*+*λ*|*z*|, where *Sλ*(*a*) represents the soft threshold operator, and the value is as follows:

(10) $$\begin{array}{c} S\lambda \left(a\right)=\left\{\begin{array}{c} a-\lambda ,a>\lambda \\ 0,\left|a\right|\leq \lambda \\ a+\lambda ,a<-\lambda . \end{array}\right. \end{array}$$

According to proposition 1, we can get the following:

(11) $$\begin{array}{c} \beta_{i}=\frac{s\lambda \overset{n}{\underset{j=1}{\sum }}{xjir}_{j}^{\left(i\right)}}{\sum_{j=1}^{n}{xi}^{2}}. \end{array}$$

Therefore, the load formula solved by the solution coordinate descent method is as follows:

(12) $$\begin{array}{c} \beta_{i}=\frac{s\lambda \left(\langle x_{i},y\rangle -\Sigma_{m}:\left|v_{m}\right|>0\langle x_{i}x_{m}\rangle v_{m}+v_{i}\right.}{x_{i}^{T}x_{i}}. \end{array}$$

Subsequently, *k* times are solved to obtain the sparse load matrix *G*=(*β*_1_, *β*_2_, ⋯, *β*_*k*_). Finally, the *k* principal components can be obtained by multiplying the obtained sparse loading matrix *G* with the original data feature matrix *Z*.

### 2.2. LSTM Model

During the model training process of LSTM, the activation function will have an impact on the model. If the network layer and the activation function are too deep and the weight is too small, it will easily lead to the disappearance of the gradient, or the phenomenon of gradient explosion will occur. For this, batch normalization (BN) is added to the model. BN uses the minibatch during learning as a unit, and normalizes the data of the node to make the mean value of 0 and the variance of 1. After normalization, scale is added to restore the data to its original state. Through BN, the learning rate of the model can be increased, and the training data can be disrupted to improve accuracy. Since it is normalized, BN will be affected by the size of the number of samples (recorded as Batch_size) selected for one training. Therefore, this experiment needs to adjust the Batch_size parameter.

In addition, the LSTM model also needs to set the number of layers of the LSTM stack, the objective function, etc. The more appropriate the parameter settings, the better the model effect and the stronger the model's predictive ability. The parameters in this experiment are set to two, the first is the number of neurons in the LSTM layer *n*, and the other is the minibatch size *m* of BN.

This paper analyzes these two parameters. In theory, the increase in the number of neurons in the hidden layer will increase the number of extracted features. The more features, the smaller the error. However, the number of neurons should not be too many, and too many will lead to overfitting.

Therefore, in this experiment, we first set the minibatch size to 0 and the number of iterations to 100. Under this condition, we sequentially increased the number of neurons one by one to analyze the change in the error value. Overall, when the number of LSTM hidden neurons is 50 and the minibatch size in the Batch_norm parameter is 40, and the RMSE and MAE values are relatively low. Therefore, when only the parameters of the LSTM model are considered, this set of parameters can be selected for model prediction.

### 2.3. CNN-LSTM Model

In this paper, SPCA is added to the traditional LSTM model for feature extraction, and a convolutional neural network (CNN, convolutional neural network) is added to the SPCA-LSTM model to extract local features. The convolutional neural network is similar to the fully connected neural network. The special feature of the network structure is that the convolutional layer and the pooling layer are added. The convolutional layer is mainly to enhance the information of the features and extract the original features. The pooling layer reduces the dimension through the operation of the pooling function.

**Figure 2:**
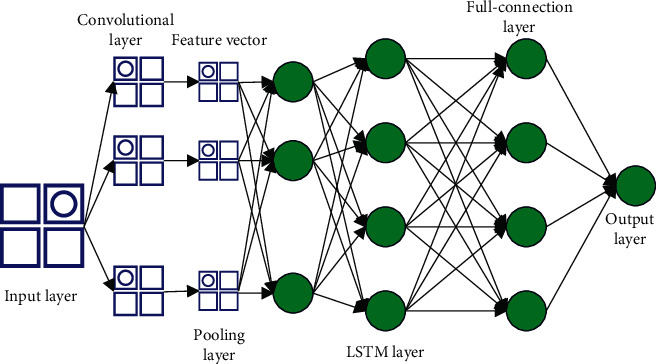
Structure diagram of CNN-LSTM network.

Based on the traditional LSTM model, the CNN-LSTM network structure diagram in this paper is shown in Figure 2, which mainly includes an input layer, a one-dimensional convolutional layer, a pooling layer, an LSTM layer, a fully connected layer, and an output layer.

**Figure 3:**
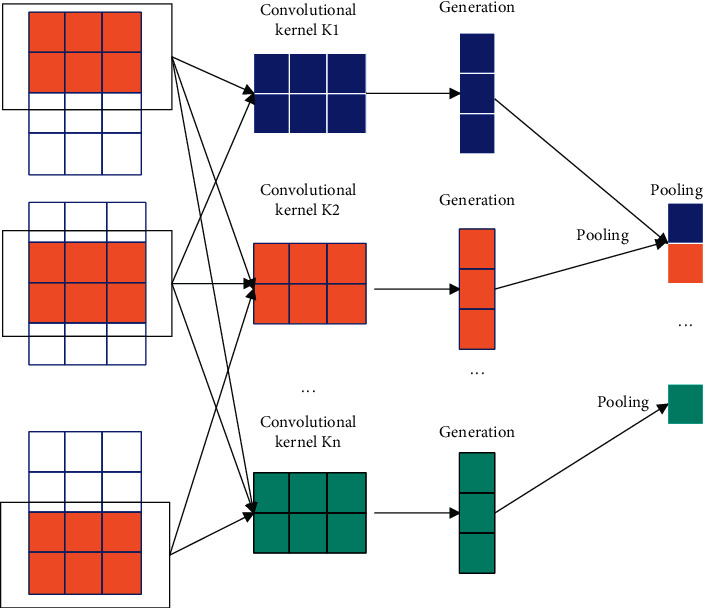
Schematic diagram of the convolution operation process.

In the convolutional layer, the traditional convolutional neural network is mainly used for image processing, and the output tensor is generated by sliding the convolution kernel in different dimensions, so as to perform three-dimensional convolutional layer operations to solve image processing problems. However, for time-series forecasting problems, due to its one-dimensional data characteristics, one-dimensional convolutional layers can be used for calculation. A schematic diagram of the operation process is shown in Figure 3.

As can be seen from Figure 3, in the process of convolution operation, the size of the convolution kernel is very important, which is related to the effect of the entire convolution operation. In general, the size setting of the convolution kernel is arbitrary. However, if the convolution kernel is too large, each operation of the convolution process will take a lot of time, resulting in low efficiency and a large amount of operation in the convolution process. If the convolution kernel is too small, only part of the features can be extracted during the feature extraction process. The resulting result is as if the hair feature of only one face image is extracted in the image processing problem, which is not conducive to image classification and may lead to wrong classification results.

In this model, the input size is assumed to be (Height, Weight), abbreviated as (H, W), the size of the convolution kernel is (FH, FW), and the size of the convolution operation is (KH, KW). The padding of the convolution process is set to P and the stride to S. Then, the KH and KW calculation formulas are as follows:

(13) $$\begin{array}{c} KH=\frac{H+2P-FH}{S}+1\\ KW=\frac{H+2P-FW}{S}+1. \end{array}$$

It can be seen from the calculation formula of KH、KW that four parameters need to be set during the convolution operation: the number of convolution kernels *n*, the size of the convolution kernel (FH, FW), the stride *S*, and the activation function *f*. At present, the commonly used activation functions are sigmoid, ReLU, etc. The ReLU function is used in this paper. The input of this model is positive, so the problem of gradient disappearance is not easy to occur, but the gradient explosion that is easy to accumulate should also be carefully accumulated during the experiment. The ReLU function expression is as follows:

(14) $$\begin{array}{c} f\left(x\right)=\left\{\begin{array}{c} x\left(x>0\right)\\ 0\left(x\leq 0\right). \end{array}\right. \end{array}$$

After the convolutional layer operation, the output vector can be expressed in the following form: output=(batch, new-steps, filters), new-steps refers to the new step size of the output, and filters represents the number of feature vectors.

Pooling is a process of abstracting information, and it is a spatial operation that reduces the amount of model parameters and optimization. The common pooling methods include average pooling and max pooling. Average pooling is to select the average value in the area, and the average value can often retain the overall characteristics of the data. Max pooling is to take out the maximum value in the target area, which tends to preserve texture features better. Therefore, average pooling is used in this experiment. The size *w* of the pooling window is usually set to the same value as the stride S.

In the LSTM layer and the fully connected layer, random orthogonal matrix initialization is used for weight initialization, and the parameter to be considered in the LSTM layer is the number of neurons *u*. The fully connected layer uses a linear activation function and is responsible for synthesizing the information. The output layer is used to output prediction results.

The loss function is calculated using mean squared error with the following formula:

(15) $$\begin{array}{c} E=\frac{1}{2}\underset{k}{\sum }{\left(y_{k}-tk\right)}^{2}. \end{array}$$

Among them, tk represents the original input data, *y*_*k*_ represents the output of the neural network, and *k* represents the dimension of the data. In the training process, batch normalization (BN) parameters are also added to each layer, and the size *m* of Batch_size needs to be determined after experiments.

## 3. Application of Improved Fuzzy Comprehensive Evaluation Method in Goodwill Evaluation and Intangible Asset Management

**Figure 4:**
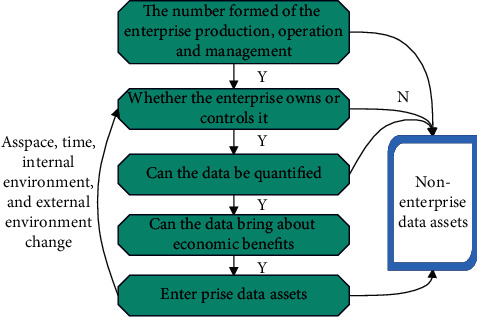
Identification process of intangible assets.

The identification process of intangible assets is shown in Figure 4.

**Figure 5:**
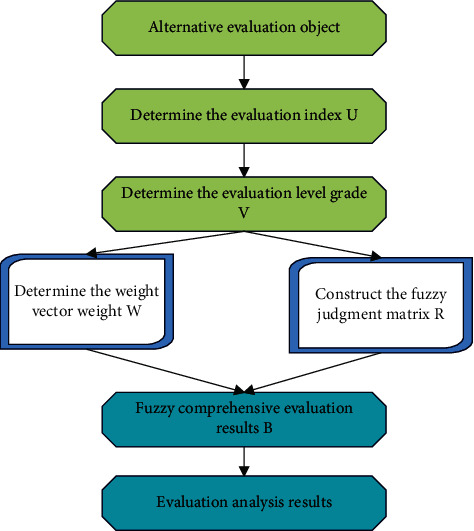
Fuzzy comprehensive evaluation method.

The fuzzy comprehensive evaluation method is applied to goodwill evaluation and intangible asset management, as shown in Figure 5.

**Figure 6:**
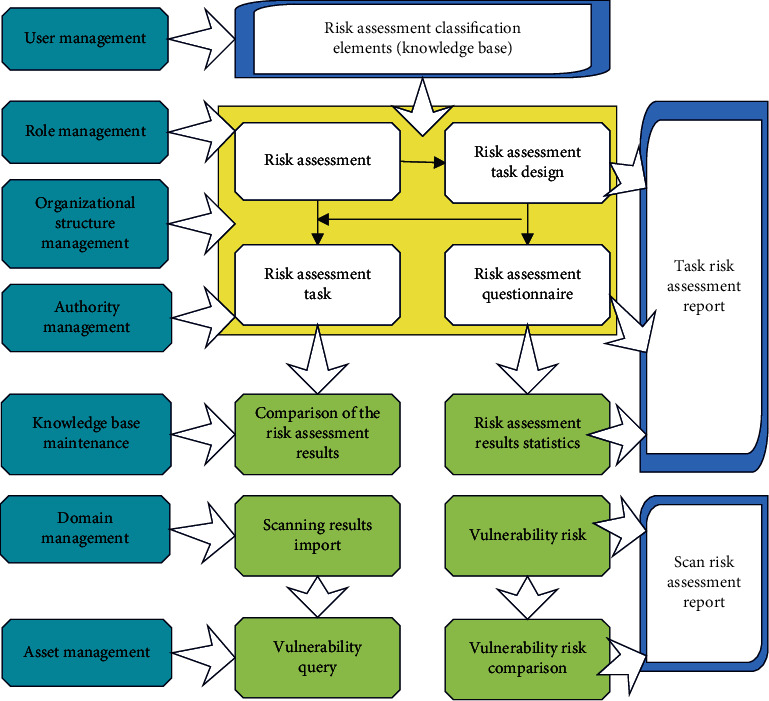
Overall design diagram of intangible asset security risk assessment.

System design work should be performed top-down. First of all, this paper designs the overall structure and then goes deeper into it layer by layer until it reaches the design of each module. The system should be supported by functions such as user management, role management, organizational structure management, authority management, knowledge source maintenance, domain management, and asset management. Through the comprehensive analysis to find out the relationship between various functions, the overall design of this system is shown in Figure 6.

**Figure 7:**
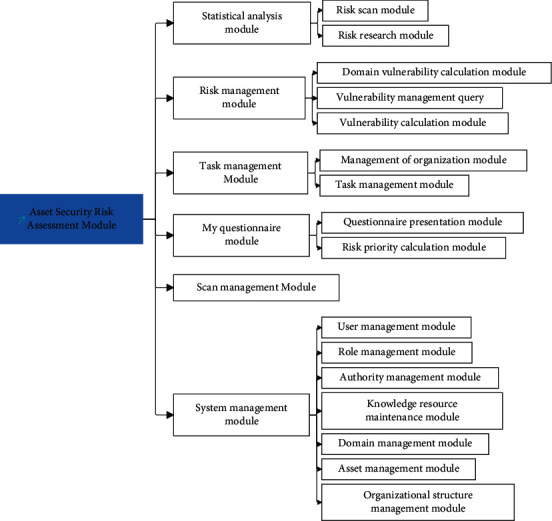
The division diagram of the asset security risk assessment module.

The basic principle of system module division according to software engineering theory is high cohesion and low coupling. The advantage of this is to facilitate the separation of module code and improve the reusability of module code. The system developed based on this principle has good maintainability and expansibility. This system uses web service technology to support the development and operation of the system under the J2EE-based environment. The system mainly includes six functional modules: system management module, scanning management module, statistical analysis module, my questionnaire module, task management module, and risk management module. The module division diagram is shown in Figure 7.

Based on the above model, the application effect of the improved fuzzy comprehensive evaluation method proposed in this paper in goodwill evaluation and intangible asset management is evaluated, and the results shown in the following Tables 1 and 2 are obtained.

It can be seen from the above research that the improved fuzzy comprehensive evaluation method proposed in this paper has a very good application effect in goodwill evaluation and intangible asset management.

## 4. Conclusions

Intangible assets are a comprehensive resource of an enterprise. In addition, intangible assets attract foreign investment to attract external investors to invest in the enterprise. The talent team, trademark, corporate culture, corporate customer list, sales channels, etc. in the enterprise are all important resources of the enterprise and must be evaluated for intangible assets. The competition of business is the competition of talents and technology. Therefore, intangible assets occupy an important position in today's business environment, which leads to intangible assets occupying an important position in today's business environment. The evaluation of intangible assets is a kind of protection and value substantiation for intangible assets. This paper combines the improved fuzzy comprehensive evaluation method to conduct research on goodwill evaluation and intangible asset management. The experimental simulation shows that the improved fuzzy comprehensive evaluation method proposed in this paper has a very good application effect in goodwill evaluation and intangible asset management.

## Figures and Tables

**Table 1:** Application effect of the improved fuzzy comprehensive evaluation method in goodwill evaluation.

Num	Goodwill assessment
1	85.41
2	83.67
3	88.69
4	81.76
5	90.81
6	90.51
7	81.32
8	86.24
9	83.36
10	86.63
11	79.40
12	88.70
13	88.27
14	88.50
15	83.07
16	87.61
17	88.03
18	79.84
19	82.16
20	85.67
21	81.15
22	81.29
23	81.07
24	90.24
25	90.42
26	88.61
27	88.65
28	80.51
29	86.53
30	79.63
31	88.26
32	80.17
33	89.56
34	82.80
35	89.70
36	84.42
37	80.03
38	81.32
39	90.04
40	81.76
41	89.96
42	88.88
43	80.42
44	87.68
45	79.22
46	79.12
47	87.09
48	89.72
49	87.89
50	85.32
51	88.03
52	90.44
53	82.75
54	80.03

**Table 2:** Application effect of the improved fuzzy comprehensive evaluation method in intangible asset management.

Num	Intangible asset management
1	85.82
2	90.38
3	84.80
4	90.01
5	87.70
6	89.43
7	92.89
8	85.80
9	90.74
10	84.89
11	91.50
12	88.80
13	84.86
14	86.96
15	91.12
16	91.08
17	90.42
18	84.24
19	90.39
20	89.45
21	85.21
22	89.45
23	89.61
24	91.16
25	92.16
26	90.87
27	92.90
28	92.62
29	89.67
30	92.99
31	90.01
32	89.88
33	92.43
34	85.58
35	90.73
36	91.32
37	92.30
38	90.17
39	92.34
40	88.36
41	89.82
42	92.52
43	89.47
44	86.86
45	92.15
46	92.44
47	84.13
48	87.57
49	88.13
50	92.31
51	84.08
52	85.24
53	86.26
54	90.28

## Data Availability

The labeled datasets used to support the findings of this study are available from the author upon request.
